# A framework for handling missing accelerometer outcome data in trials

**DOI:** 10.1186/s13063-021-05284-8

**Published:** 2021-06-05

**Authors:** Mia S. Tackney, Derek G. Cook, Daniel Stahl, Khalida Ismail, Elizabeth Williamson, James Carpenter

**Affiliations:** 1 Department of Medical Statistics, London School of Hygiene and Tropical Medicine, London, UK; 2 Population Health Research Institute, St George’s, University of London, London, UK; 3 MRC Clinical Trials Unit, University College London, London, UK; 4 Department of Biostatistics & Health Informatics, King’s College London, London, UK; 5 Department of Psychological Medicine, King’s College London, London, UK

**Keywords:** Clinical trial, Accelerometer, Wearables, Missing data, Multiple imputation

## Abstract

Accelerometers and other wearable devices are increasingly being used in clinical trials to provide an objective measure of the impact of an intervention on physical activity. Missing data are ubiquitous in this setting, typically for one of two reasons: patients may not wear the device as per protocol, and/or the device may fail to collect data (e.g. flat battery, water damage). However, it is not always possible to distinguish whether the participant stopped wearing the device, or if the participant is wearing the device but staying still. Further, a lack of consensus in the literature on how to aggregate the data before analysis (hourly, daily, weekly) leads to a lack of consensus in how to define a “missing” outcome. Different trials have adopted different definitions (ranging from having insufficient step counts in a day, through to missing a certain number of days in a week). We propose an analysis framework that uses wear time to define missingness on the epoch and day level, and propose a multiple imputation approach, at the day level, which treats partially observed daily step counts as right censored. This flexible approach allows the inclusion of auxiliary variables, and is consistent with almost all the primary analysis models described in the literature, and readily allows sensitivity analysis (to the missing at random assumption) to be performed. Having presented our framework, we illustrate its application to the analysis of the 2019 MOVE-IT trial of motivational interviewing to increase exercise.

**Supplementary Information:**

The online version contains supplementary material available at (10.1186/s13063-021-05284-8).

## Introduction

The use of pedometers, accelerometers and other devices to measure physical activity in clinical trials has increased rapidly in the last 20 years [1]. These tracking devices offer step count and intensity of activity measurements in very fine intervals of time, often in seconds, which removes recall and desirability bias that come with measuring activity using self-report approaches [2]. A number of clinical trials have used accelerometers to measure change in participants’ physical activity for a period of 7 days before and after an intervention, possibly with additional follow-up. The MOVE-IT trial measured participants’ activity for 7 days on three occasions: at baseline, after implementing group or individual motivational therapy, and one year after the completion of therapy [3]. Other trials with a similar structure in measurement period and follow-up include the PACE-UP and PACE-LIFT trials, which measured participants’ activity for 7 days before and after implementation of a walking-based intervention [4–6]. In the Trial of Activity in Adolescent Girls (TAAG), effect of school- and community-based interventions on physical activity in middle-school girls was measured by accelerometers [7, 8]. A similar set-up was used in studies investigating telephone-delivered interventions on lung cancer patients [9] and diabetic patients [10].

The proportion of planned data that each participant contributes varies due to a number of factors. Participants may forget to wear the accelerometer and provide reduced step counts for some days, and there are also protocol-compliant reasons for removing the accelerometer, such when swimming or showering if the accelerometer is not water proof [7]. There may also be technical issues such as the battery running out, or the device failing if used accidentally under water. Despite the ubiquity of missing data in trials using accelerometer outcome data, there is currently little guidance on how to handle this in the analysis. At a very fundamental level, there is currently no consensus on the definition of missing data in this context [2]. Accelerometers record accelerations in three dimensions over very fine intervals of time, called epochs. These accelerations can be processed to calculate the number of steps taken. If zero accelerations are registered for a particular epoch, and especially for a large number of adjacent epochs, it is not obvious whether the participant is wearing the device and simply staying still, or whether the device has been removed and no data is being collected. When focusing on daily step counts, if a participant does not wear the accelerometer for the entire day, this is clearly an instance of missing data; less clear-cut is defining what a sufficient amount of wear time of the accelerometer should be, in order for the observation to be deemed a complete daily step count observation. There are many suggestions in the literature for how to define missingness in terms of a threshold for daily step count or wear time [10–12]. Further, if step counts from an insufficient amount of time are provided, there is the question of whether this data should be considered as censored data, or whether those counts should be discarded and that day should be considered missing. The two approaches lead to different requirements in the analysis. The combination of increased use of accelerometer data and a lack of guidance in how to handle missing data in this context means that there is a need to evaluate the various possible approaches to handling missing data analysis and then derive a framework which can be used to inform future trial designs, protocols and statistical analysis plans.

If there are no missing data, analysis of repeated step counts is typically conducted by a simple linear regression of step counts aggregated over the measurement period. Multilevel modelling is also often useful when the study is longitudinal or if there is clustering of participants. A primary analysis of the trial generally has average step counts over the measurement period (e.g. average daily step counts over a pre-specified week) as the outcome, which allows a straightforward comparison of average step counts pre- and post-intervention. In contrast to daily step counts, which are bounded at zero and typically mildly skewed, average step counts over the measurement period are likely to be approximately normal, making an analysis on the untransformed scale sufficient. Supplementary analysis could include a mixed model with daily step counts as the outcome, which allows for the study of day-level variables such as day of the week or weather.

When there are missing data, there are two commonly used principled approaches of handling them. Firstly, missing outcome data are easily handled by maximum likelihood methods for linear regression or mixed models under the missing at random (MAR) assumption [13, p. 56]. Under the MAR assumption, missingness depends on the observed step counts and/or covariates included in the primary analysis model; given these, it does not depend on the unobserved pattern of step counts [7]. Now, a key issue is that the MAR assumption may be implausible in the accelerometer setting since it is quite likely that participants do not wear their device during days or parts of days where they are less active; an analysis that assumes that data are MAR when it is not can lead to invalid estimates.

Alternatively, multiple imputation (MI) offers a flexible approach to the analysis of accelerometer data with missing values. An imputation model is specified, which is a model for the posterior predictive distribution of the missing outcomes given the observed data; this is used to impute the missing outcomes with plausible values, taking full account of the uncertainty [14]. A total of *M* sets of complete data are constructed and the analysis model is fitted to each of them. The results of the *M* analysis models are combined using Rubin’s rules to incorporate uncertainty due to the missing data [15]. The imputation model and the analysis model are separate; this feature is particularly appropriate in the accelerometer setting where the primary analysis model may have averaged step counts as the outcome, but missingness is defined for smaller intervals of time, most typically for days, so constructing the imputation model with daily step counts as the outcome is a natural approach. It is possible to incorporate step counts as right-censored observations with MI if participants took an insufficient number of steps. MI typically assumes MAR given the variables included in the imputation model; the imputation model may include additional auxiliary variables, such as daily weather variables, to make the MAR assumption more plausible. Further, MI also allows for the relatively simple implementation of sensitivity analysis to assess the robustness of the results to the untestable missing data assumptions [16]; see Harris et. al. (2017) for an example of a sensitivity analysis on the possible impact of step counts being MNAR [5].

In this paper, we propose a framework for handling missingness in accelerometer outcome data. This involves aggregating step counts on the day level, and classifying them into missing, partially observed, and observed step counts, and identifying appropriate assumptions for the missing and partially observed data. Then, MI can be used to impute daily step counts; the imputed datasets can be analysed to take into account the uncertainty due to the missing data. We implement this framework in an analysis of the MOVE-IT trial. In the “Motivating Example: MOVE-IT trial” section, we introduce the MOVE-IT trial, and we describe the primary analysis typically conducted in accelerometer trials in “Primary analysis.” In the “What constitutes a missing step count?” section, we explore the definition of missing data in the accelerometer setting. We then elucidate our framework for MI in the “Framework for handling missing data” section, where we set out our recommendations for classifying daily step counts into observed, partially observed and missing data, where assumptions need to be specified for the missing and partially missing data. We outline the need to include auxiliary variables to strengthen the MAR assumption, and we outline how MI can be conducted with Tobit regression to account for partially observed data. In the “Applying the framework to the MOVE-IT trial” section, we illustrate the approach with data from the MOVE-IT trial and display the results. Finally, there is a “Discussion” section.

## Motivating Example: MOVE-IT trial

Throughout this paper, we use data from the MOVE-IT trial to demonstrate the decisions needed in conducting an analysis of accelerometer data with MI, and associated analysis results. The trial investigated the effects of motivational interviewing and motivational group therapy for patients at high risk of cardiovascular disease (QRISK2 of 20% or higher [17]) in reducing weight and increasing physical activity. The trial randomized patients to one of the following: individual motivational interviewing, motivational group therapy, or usual care. Individual motivational interviewing and motivational group therapy consisted of ten sessions over the course of a year. Between June 2013 and February 2015, 1742 participants were recruited from 135 general practices across the 12 South London Clinical Commissioning Groups. We focus on step count as a measure of physical activity. The participants were provided with an ActiGraph GT3X accelerometer (ActiGraph, FL, USA) for a period of seven consecutive days on three occasions: baseline, 12 months, and 24 months. The protocol and results of the trial have been reported previously [3, 18, 19]. There was insufficient evidence from the trial to recommend motivational interviewing or motivational group therapy for reducing weight or increasing physical activity.

## Primary analysis

**Fig. 1:**
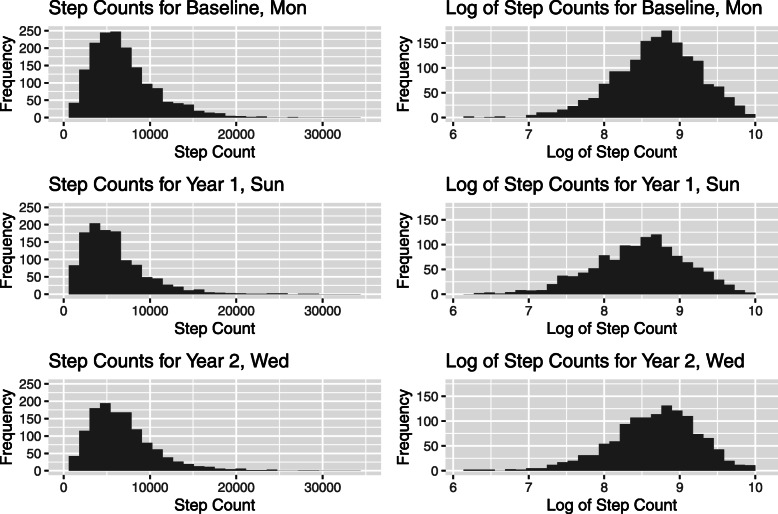
Histograms of step counts for Monday at baseline, Sunday at year 1 and Wednesday at year 2 (left) and histograms of the logged step counts (right)

We assume that, in common with many clinical trials, the primary analysis investigates whether the intervention led to any change in step count as a measure of physical activity between baseline and post-intervention measurements. The outcome is step count averaged over the measurement period, and if there is more than one post-intervention measurement, a multilevel analysis is typically needed to account for the clustering of observations per participant ID. An advantage of using averaged step count as the outcome is that a model on the untransformed scale is likely to fit reasonably well. Daily step counts are generally more left-skewed than averaged step counts. For example, in the MOVE-IT trial data, Fig. 1 shows histograms of step counts for a randomly chosen day within each measurement period: Monday for baseline, Sunday for year 1 and Wednesday for year 2 on the left, and the log of those counts on the right. If step counts are analysed at the daily level, then a log transformation is likely to be needed if the analysis relies on multivariate normality, which will in turn have implications both for the interpretation of the analysis and for the imputation model.

We assume that the average step count across the measurement period is our outcome. To illustrate a possible primary analysis using the MOVE-IT trial as an example, suppose that *y*_*i,j,k*,*k*_ is the step count for patient *i* at year *j* on day *k*, where *i*∈{1,...,1742},*j*∈{0,1,2} and *k*={1,2,...,7}. We define $\bar {y}_{i,j,.}$ as the average step count across seven consecutive days for patient *i* at year *j*:

1 $$ \bar{y}_{i,j,.}=\frac{1}{7}\sum_{k=1}^{7} y_{i,j,k},  $$

and we denote by arm_*i*_ the arm that participant *i* was assigned to, which takes values 1, 2 or 3, corresponding to individual therapy, group therapy and usual care, respectively. In the MOVE-IT trial, in the absence of missing data, the pre-specified primary analysis model has a baseline-year interaction, a treatment-year interaction, and fixed effects for sex, age and borough, so can be written, for *j*∈{1,2}:

2 $$ {\displaystyle \begin{array}{cc}{\bar{y}}_{i,j,.}& ={\beta}_0+{\beta}_1\mathrm{year}\ {2}_{i,j}+{\beta}_2\mathrm{I}\left({\mathrm{arm}}_i=1\right)+{\beta}_3\mathrm{I}\left({\mathrm{arm}}_i=2\right)\\ {}+{\beta}_4\mathrm{year}\ {2}_{i,j}\times \mathrm{I}\left({\mathrm{arm}}_i=1\right)+{\beta}_5{\mathrm{year}2}_{i,j}\\ {}\times \mathrm{I}\left({\mathrm{arm}}_i=2\right)\\ {}+{\beta}_6{\bar{y}}_{i,0,.}+{\beta}_7\mathrm{year}\ {2}_{i,j}\times {\bar{y}}_{i,0,.}+{\beta}_8{\mathrm{female}}_i+{\beta}_9{\mathrm{age}}_i\\ {}+{\beta}_{10}b{1}_i+{\beta}_{11}b{2}_i+\dots +{\beta}_{21}b1{1}_i+{e}_{i,j},\end{array}} $$

where covariates in the model include:

- year 2, the dummy variable for whether the observation is from year 2;
- female, the dummy variable for whether the participant is female;
- age, the age of the participant at baseline in years (centred);
- *b*1,*b*2,...,*b*10,*b*11, dummy variables for the borough of London that the participant resides in.

To take account of the effect of the intervention on the variance, the primary analysis also specifies an unstructured correlation matrix for the residuals by arm:

3 $$ \left(\begin{array}{l} e_{i, 1}, e_{i, 2} \end{array}\right)^{\top} \sim N \left(0, \Sigma_{\text{arm}_{i}} \right),  $$

where the variance-covariance matrix is given by

4 $$ \Sigma_{\text{arm}_{i}} = \left[\begin{array}{ll} \sigma_{1, \text{arm}_{i}}^{2} & \sigma_{12, \text{arm}_{i}} \\ \sigma_{21, \text{arm}_{i}} & \sigma_{2, \text{arm}_{i}}^{2} \end{array}\right],  $$

where $\sigma _{j, \text {arm}_{i}}^{2}$ denotes the variance of residuals for year *j* for patients assigned to arm_*i*_, and $\phantom {\dot {i}\!}\sigma _{12, \text {arm}_{i}}=\sigma _{21, \text {arm}_{i}}$ denotes the covariance between residuals at year 1 and year 2 for patients assigned to arm_*i*_.

In this trial design, patient outcomes in the group therapy arm may also be correlated because of a common therapy group effect. We investigated including a random effect to model this, but there appeared to be insufficient information in the data to support this, causing the model not to converge. Working with this primary analysis model, our goal is to handle the missing data appropriately. This requires, first, a discussion of what constitutes missing data in the accelerometer setting.

## What constitutes a missing step count?

Accelerometers measure acceleration in three dimensions at the epoch level, which is typically set to 5- or 10-s intervals. These are then converted to step counts over the epoch. There are two main issues associated with defining missing accelerometer data. Firstly, it is difficult to distinguish between periods where participants are wearing the device as per protocol but are inactive, and periods where data is missing as the participant has stopped wearing the device. In both instances, the accelerometer will register zero step counts over the epochs of that time period. Secondly, step counts at the epoch level are generally aggregated at a higher level, usually at the day level. Missingness then needs to be defined at this higher level.

Measuring the *weartime* of the accelerometer is important in addressing both of these issues. Wear time is the length of time that the device is worn per day. To address the first issue, the accelerometer is typically set to ignore long runs of epochs with zero step counts, where a long run is often defined to be a period that is longer than 20, 40 or 60 min [20]. Thus, during long periods where no acceleration is detected by the device, it is assumed that the participant has stopped wearing the device and the epochs within such periods are instances of missing data. More sophisticated methods of defining wear time are continually being developed; see, for example, Syed et al. (2020) [21].

To address the second issue, most authors impose a cut-off at the day level for the number of steps taken or wear time, where days that do not achieve the cut-off are viewed as a missing observation, and any step counts collected that day are discarded. For example, De Craemer et al. (2016) require a minimum of 6 h of wear time [22], while some other authors require more than 10 h of wear time per day [9, 23]. Some authors impose an additional cut-off on the number of missing days in the measurement period, and the entire period is regarded as missing if this condition is not met. For example, Bade et al. (2018) consider data from a participant to be missing if less than 200 steps were taken per day or if data for less than 5 days were collected per week [10]. Kloeck et al. (2018) state that data from a participant across the measurement period is valid if patients wore the device for ≥3 days, for ≥8 h per day [12]. Kipping et al. (2014) require that participants have 3 days (out of five) with at least 8 h of wear to be considered valid [11].

Our recommendation is to aggregate data on the day level, which appears natural as participants are typically provided with instructions on how to wear the device over the course of a day (for example, to wear between waking and sleeping, except when bathing or swimming). We then recommend imposing a cut-off in terms of wear time to distinguish between observed step counts and partially observed step counts. Thus we define a daily step count value as observed, $y_{i,j,k}^{\text {obs}}$, when wear time is greater than the cut-off value. A step count value is partially observed, $y_{i,j,k}^{\text {partial}}$, if wear time is between zero and the cut-off (exclusive). A missing step count, $y_{i,j,k}^{\text {mis}}$, occurs when the device is not worn and the wear time is zero. Where participants wear the device but provide an insufficient amount of time for a particular day, the step counts of that day are treated as right-censored data, rather than discarding this information as missing. The cut-off value should be explicated in the trial protocol. While total step counts are often of interest in exercise trials, we note that this framework is applicable more generally. For example, if there is interest in the number of steps taken over a specific part of the day, it is equally possible to define observed, partially observed and missing step counts in an analogous way.

Though participants are typically required to provide step counts for consecutive days over a measurement period, sometimes, participants may provide data for days before or after the measurement period if they wore the accelerometer for longer than necessary. If we can assume that step counts of a particular participant are exchangeable across adjacent weeks, given the day of the week, we can substitute missing daily step count $y_{i,j,k}^{\text {mis}}$ with an observed step count $y_{i,j,k}^{\text {obs}}$ from day *k* of the following week, or it can be substituted with a partially observed step count $y_{i,j,k}^{\text {partial}}$ from the following week if no observed step count is available. Further, a partially observed step count $y_{i,j,k}^{\text {partial}}$ from the measurement period can be substituted with an observed step count for day *k* from the following week. For example, if a participant’s step count for Tuesday is missing during the 7-day measurement period, but there is either an observed or partially observed step count for the following Tuesday, the observed or partially observed step count would be used in the analysis. We refer to this as *day-substitution*. Where the assumption of exchangeability across weeks, given the day of the week, is plausible, day-substitution allows for a greater amount of information from the data collection to be retained for the analysis. Day-substitution would not be appropriate if, for example, participants chose not to wear the accelerometer during the first week because they were less active and tried to compensate by being more active the following week. It is thus crucial to consider whether the distribution from which the later step count is drawn is the same as that from which the missing step count is drawn. Ideally, day-substitution should be an a priori decision built into the protocol where participants are instructed not to wear the accelerometer for more than seven full days; where there are days with insufficient wear, the participant is asked to wear the accelerometer in the following week, on the same day of the week.

**Fig. 2:**
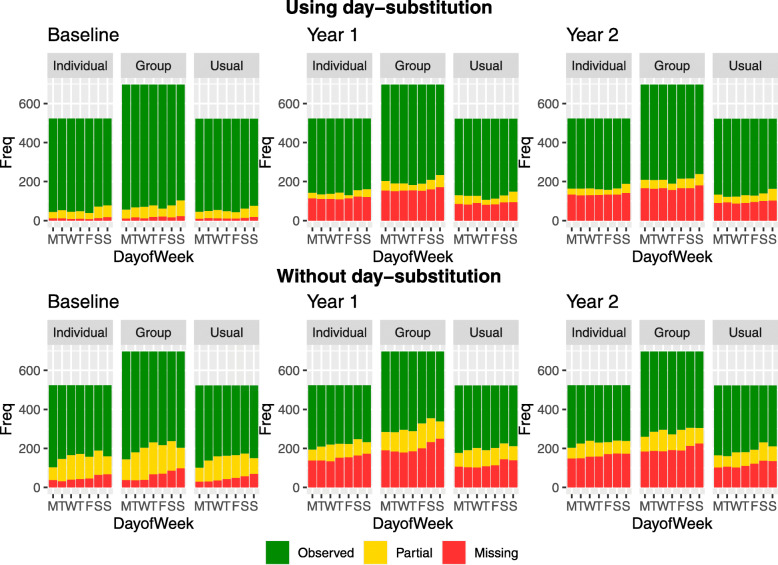
Frequency of daily step count observations for each day of the week at baseline (left), year 1 (center) and year 2 (right) classified as observed (green), partially observed (yellow), and missing (red) when day-substitution is used (top row) and when day-substitution is not used (bottom row)

In the MOVE-IT trial, participants were told to wear the accelerometer all day between waking up and sleeping, removing the device only when bathing or swimming. Runs of zero counts lasting 60 min or longer were ignored when calculating wear time. Daily step counts were deemed observed when wear time is greater than 540 min, which was specified in the protocol. According to the protocol, if participants failed to wear the device for at least 540 min on each of at least 5 days at baseline, they were asked to wear the accelerometer for another 7 days [19]. This was also done in the PACE-LIFT trial [4]. This particular practice reduces missingness at baseline. However, only 2.35*%* of patients provided data with wear time of greater than 540 min for seven consecutive days at baseline, year 1 and year 2, demonstrating the ubiquity of missing data. A further complication is that, several participants wore the accelerometer for more than 7 days. Figure 2 displays the number of observed, partially observed and missing observations for each combination of day of the week, arm and year when day-substitution is carried out in the top row, and when it is not carried out in the bottom row. We observe that day-substitution leads to slightly fewer missing and partially observed values. We note that there is a greater number of observations in the group therapy arm as the recruitment ratio was 3:4:3 for the individual therapy, group therapy and usual care arms, respectively.

**Fig. 3:**
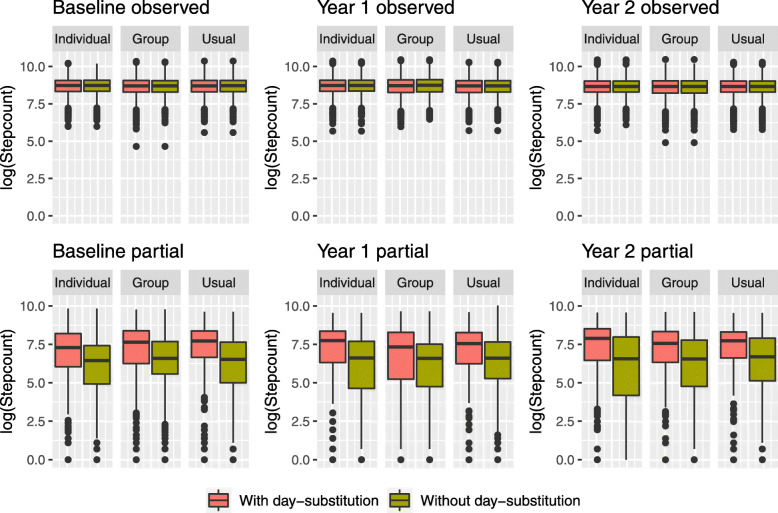
Boxplots showing the distribution of the log of observed daily step counts (top) and log of partially observed step counts (bottom) at baseline, year 1 and year 2 when day-substitution is carried out across weeks (red) and when it is not carried out (green)

In the MOVE-IT trial, using day-substitution affects not only the proportion of missing and partially observed step counts, but it also affects the distribution of step counts. Figure 3 displays boxplots showing the distribution of logged step counts for complete observations (wear time ≥540), and that of partial observations (0< wear time <540) in the bottom row for each year when day-substitution is carried out (in red) and when it is not carried out (in green). For observed step counts, we see that day-substitution makes very little change to the distributions. For the partially observed step counts, we observe that the median of the boxplots are higher when day-substitution is carried out. For partially observed step counts, it appears that day-substitution leads to a higher number of steps. This is due to the fact that participants have increased wear time on substitute days; a visualization of the distribution of wear time in the “Wear time” section displays a similar pattern to Fig. 3. This suggests that the exchangeability assumption across weeks given the day of the week may not be plausible in this setting. Day-substitution based on a criterion other than day of the week may be more appropriate. For example, in the “Auxiliary variables” section, we show that the level of sunshine during the day is predictive of missingness and also of step count. A missing step count in the measurement period could be substituted with a step count from a day outside of the measurement period with a similar level of sunshine.

## Framework for handling missing data

Analysis of data with missing values requires assumptions about the missingness mechanism. A popular categorization of these mechanisms was provided by Rubin (1976) [15]. Under the assumption that the data are missing completely at random (MCAR), the probability that an observation is missing is independent of the observed or unobserved data. The assumption that the data are missing at random (MAR) states that the probability that an observation is missing is independent of the unobserved data, conditional on the observed data. In our setting, this means that, within groups of step count outcomes defined by the observed covariates, the probability of an observation being missing is the same. Finally, the assumption that the data are missing not at random (MNAR) states that the probability that an observation is missing depends on the unobserved data. Inference under MNAR requires a specification of how the conditional distributions of the step counts given the observed covariates differ for those participants who do, and those do not, have missing outcomes [24, p. 17]. It is not possible to prove that the MAR assumption is met using only the observed data, so sensitivity analyses are needed to understand the robustness of the results to the MAR assumption [25].

In this framework for handling missing step counts, we define missing data on the epoch level as a long run of zeros, typically set to 60 min. We then aggregate the step counts at the day level, as there are several advantages for doing so. Firstly, as mentioned in the “What constitutes a missing step count?” section, it may be possible in some cases to substitute missing or partially observed step counts with step counts from outside the measurement period on the same day of the week. This allows for a greater proportion of collected data to be retained for the analysis. Secondly, partially observed daily step counts can be incorporated in the imputation model. Partially observed data can be regarded as right-censored data, as the true step count is higher than that observed if the participant prematurely stopped wearing the accelerometer; this can be incorporated in the imputation model though Tobit regression. Thirdly, while step counts averaged across the measurement period are likely to be MNAR, since participants may be more vigilant about wearing the accelerometer during periods when they are active, and may remove the device during sedentary periods, the assumption can be made more plausible for daily step counts by the inclusion of auxiliary variables such as day of the week or weather variables. The four key decisions in our framework for obtaining an appropriate imputation model for daily step count data are as follows:

1. Whether to use *day-substitution*. The assumption of exchangeability of step counts across weeks can be assessed by inspecting the distributions of the observed and partially observed step counts when day-substitution is used, and when it is not, as shown in Fig. 3. If there is evidence that participants who wear the accelerometer outside of the measurement period are doing so because they are more physically active outside of the measurement period, this assumption would incur bias.
2. Whether to impute $y_{i,j,k}^{\text {partial}}$ as *censored data* or to impute them as missing data. If they are imputed as censored data, this means that $y_{i,j,k}^{\text {partial}}$ is the lower bound for the number of steps that the participant took that day; incorporating this leads to a slightly more complicated analysis, which can be achieved using MI. It may be that, for some days, participants fall short of the threshold for a complete observation by a small margin; in this case, partially observed data provide a lot of information about the participants’ activity so there is a strong case for incorporating partially observed data.
3. Whether to *assume MAR* for $y_{i,j,k}^{\text {mis}}$ and/or $y_{i,j,k}^{\text {partial}}$. Under the MAR assumption, the expected number of steps that participants take when they wear the accelerometer is the same as the expected number of steps that they take when they forgo wearing the accelerometer, conditional on all other variables. If participants are more likely to wear it on active days compared to inactive days, these assumptions would be inappropriate. Using sensitivity analysis via MI, it is possible to explore scenarios where step counts are missing not at random, for example, where participants’ propensity to exercise is reduced in the periods where they are not wearing the accelerometer.
4. Whether to use *auxiliary variables*. If the MAR assumption is made above, the addition of auxiliary variables may strengthen its plausibility. This is discussed in more detail in the “Auxiliary variables” section.

Five possible approaches to the decisions outlined above are given in Table 1, which correspond to the primary analysis, the key sensitivity analysis, and three possible additional sensitivity analyses:

- *Plausible*: This is our recommended model for the primary analysis. Day-substitution is not carried out, and $y_{i,j,k}^{\text {partial}}$ are imputed as censored. We assume that $y_{i,j,k}^{\text {mis}}$ and $y_{i,j,k}^{\text {partial}}$ are MAR given variables in the imputation/analysis model, and auxiliary variables are included in the imputation model.

**Table 1:** Framework for assumptions on missing data

	Primary	Key sensitivity	Other sensitivity nalyses:		
	analysis:	analysis:			
**Decisions**	*Plausible*	*Suspicious*	*Plausible-no-aux*	*Replace-days*	*Dismissive*
**Use day-substitution?**	✗	✗	✗	✓	✗
**Impute as censored?**	✓	✓	✓	✓	✗
**Assume MAR?**	✓	✗	✓	✓	✗
**Use auxiliary variables?**	✓	✓	✗	✓	✓
- *Suspicious*: This is our recommended model for the sensitivity analysis. As in the *Plausible* model, day-substitution is not carried out, and $y_{i,j,k}^{\text {partial}}$ are imputed as censored. However, we explore in this analysis the sensitivity to departures from the MAR assumption. Under this model, we assume that the step counts are likely to be lower if participants have removed their accelerometer by a proportion *δ*. Auxiliary variables are included.
- *Plausible-no-aux*: The *Plausible* model with auxiliary variables omitted.
- *Replace-days*: Day-substitution is carried out assuming that participants’ step counts are exchangeable across weeks, given the day of the week. Then, $y_{i,j,k}^{\text {partial}}$ are imputed as censored and $y_{i,j,k}^{\text {mis}}$ and $y_{i,j,k}^{\text {partial}}$ are assumed to be MAR given the variables in the imputation/analysis model. Auxiliary variables are included in the imputation model.
- *Dismissive*: Day-substitution is not carried out. Further, if we have partially observed data $y_{i,j,k}^{\text {partial}}$, they are discarded and treated as $y_{i,j,k}^{\text {mis}}$. We assume that $y_{i,j,k}^{\text {mis}}$ are MNAR. We assume that the log of step counts are lower by a proportion *δ* if participants removed their accelerometer for the entire day. Auxiliary variables are included.

### Auxiliary variables

An advantage of using MI is that it is possible to incorporate auxiliary variables into the imputation model which makes the MAR assumption more plausible. Auxiliary variables are variables which are predictive of missingness of the outcome and also of the value of the outcome [24, p. 64]. They are not conditioned on in the primary analysis; the advantage of using MI is the separation of the imputation and analysis models which allows for an imputation model where the MAR assumption can be made more plausible.

For example, in the MOVE-IT trial, Body Mass Index (BMI) at baseline was found to be predictive of both average step count and missingness at the week level at the 5% level of significance. Further, we considered weather variables as potential auxiliary variables. A number of longitudinal studies have demonstrated a link between weather and physical activity; activity has been shown to decrease with increased rainfall [26–28]. When temperature diverges from the average climate temperature, physical activity has been shown to decrease [26, 28, 29] and increased daylight hours has been shown to be associated with increased physical activity [30]. In the MOVE-IT trial, the following weather variables were individually found to be predictive of both daily step count values and missingness at the 5% level: maximum air temperature during the day between 9AM and 9PM (temperature), the square root of the total rainfall per day for the 24-h period in millimetres (rainfall), sunshine duration in hours (sunshine) and daylength in hours (daylength). We therefore include BMI at baseline and these weather variables as auxiliary variables in our analysis. In the “Obtaining weather data” section, we describe how we obtained the weather data. The MAR assumption may be more plausible after accounting for the participants’ BMI at baseline and day-level weather variables, in addition to variables included in the primary analysis.

### Imputation

After defining a model for the primary analysis such as in Equation 2, defining missingness in the context of accelerometer data and constructing a set of possible assumptions for the missing data such as in Table 1, the next step is to identify an approach for imputing the missing data under those assumptions. Some analyses of accelerometer data with missing observations have involved imputation using the Expectation-Maximization (EM) algorithm. Catellier et al. (2005) showed in a simulation that single imputation using the EM algorithm and multiple imputation had similar performance in term of bias and precision [7]. In general, we do not recommend this approach as single imputation does not take full account of the uncertainty. We recommend the use of multiple imputation (MI) to handle missing data in this context as it allows for:

1. The separation of the imputation and analysis models so that the imputation model can have step counts on the day level and the analysis model can have step counts on the week level;
2. The incorporation of partially observed (bounded below) step count data with Tobit imputation;
3. The addition of auxiliary variables;
4. The relatively simple use of sensitivity analysis to explore a range of missingness assumptions.

### Tobit regression

For our models which incorporate partially observed daily step counts, Tobit imputation can be used. Tobit regression is a method for estimating linear relationships between variables when the outcome is left- or right-censored [31, 32]; in our setting, the accelerometer outcome is right-censored for participants who wear the device for an insufficient amount of time. Tobit regression can be implemented using interval regression in STATA, which requires specification of lower and/or upper bounds for each observation. For completely missing daily step counts, the lower bound is zero, and for partially observed daily step counts, the lower bound is the recorded step count. The upper bound can be set to a limit chosen to be greater than any observed daily step count. For complete observations, the lower and upper bound are the recorded value. Thus the imputation model is specified such that draws from the posterior predictive distribution of the missing values given the observed data are bounded between the recorded value and the chosen upper limit for partially observed step counts, and bounded between zero and the chosen upper limit for missing step counts.

### Software

Currently, the only available Tobit imputation routine which combines the resulting imputed datasets for a mixed model primary analysis with a complex residual structure is in STATA with chained equations: mi impute chained (intreg). It was originally written for the ice macro [33]. We provide example code in Additional file 1.

A recent R package hmi [34] is able to handle multiple imputation for interval data and can be used if the primary analysis is a linear regression or a multilevel model with homoscedastic residuals. As it currently only allows for homoscedastic residuals, it would not be appropriate in the analysis of the MOVE-IT trial where the primary analysis has a complex residual structure. The extension of other R packages for multiple imputation, such as jomo [35], to incorporate routines for the interval regression setting is a possible avenue for further exploration.

## Applying the framework to the MOVE-IT trial

We now describe how the imputation model is specified for the MOVE-IT trial for the five possible assumptions in Table 1. Our imputation model has the log of the step count as the outcome; this allows us to impute missing step counts conditional on other days of the week that year, as well as days from the other 2 years, and important auxiliary variables. After imputation, the *M* sets of logged step counts are then exponentiated to obtain daily step counts, and averaged across the 7-day measurement period in order to obtain week-average step counts for the analysis model.

The *Replace-days* and *Plausible* assumptions for the missing data incorporate partially observed data $\log y_{i,j,k}^{\text {partial}}$ into the analysis, so we use Tobit regression. We set the lower bound *l*_*i,j,k*,*k*_ as $\log y_{i,j,k}^{\text {partial}}$ and we set the upper bound *u*_*i,j,k*,*k*_ at a value that is at least as high as the log of the highest step count observed in the study. We note that the *Suspicious* and *Dismissive* assumptions for the missing data, which do not incorporate partially observed data, could be handled using linear regression instead of Tobit regression. However, using linear regression can lead to imputed values which are much higher than the upper bound *u*_*i,j,k*,*k*_, which in turn leads to an unrealistically high step count when exponentiated. To keep the results under the five sets of assumptions for the missing data comparable, we use Tobit regression for all four imputation models where the upper bound for missing or partially observed log counts are set to *u*_*i,j,k*_.

We explore the possibility that the step counts are missing not at random in the *Suspicious* and *Dismissive* assumptions for the missing data, where we assume that participants’ propensity to exercise is reduced in the periods where they are not wearing the accelerometer. We assume that, under MNAR, $\log y_{i,j,k}^{\text {partial}}$ is 0.95 times that obtained under the MAR assumption. Similarly for missing data, under MNAR, we assume that $\log y_{i,j,k}^{\text {missing}}$ is 0.95 times that obtained under the MAR assumption. This means that, after MI under the MAR assumption, we simply multiply the resulting log step counts which were missing or partially missing in the study by 0.95. The assumption that step counts are 5% less than that expected under MAR on the log scale implies that, on the step count scale, a greater expected step count under MAR means that a greater reduction of steps is needed to obtain the expected step count under MNAR. An expected step count of 30000 under MAR is exp(log(30,000)×0.95)=17917 under MNAR, and an expected step count of 500 under MAR is exp(log(500)×0.95)=366.45 under MNAR.

We impute separately for each arm. We denote by $\log y_{i,j, k}^{l}$ the log of the step count for participant *i* in year *j* on day *k* with the superscript *l*=arm_*i*_. Further, we denote by $\overline { \log (y_{i,j,.}^{l})}$ the average of the seven logged step counts for participant *i* in year *j*:

5 $$ \overline{ \log (y_{i,j,.}^{l})}=\frac{1}{7}\sum_{k=1}^{7} \log y_{i,j,k}^{l}.  $$

The baseline log step counts $ \log y_{i,0, q}^{l}$ are imputed conditional on the baseline log step counts from other days of the week, the log step counts from year 1 and log step counts from year 2, as well as the variables of interest in the analysis model and the additional auxiliary variables. The mean of the baseline log step counts is updated as a result of this imputation. Then, the log step counts for year 1, $\log (y_{i,1,k}^{l})$ are imputed conditional on the mean of the baseline log step counts, the log the year 1 step counts from other days of the week, and the log of the year 2 step counts. The year 1 daily log step count is updated. Finally, the log step counts for year 2, $\log (y_{i,2,k}^{l})$ are imputed conditional on the mean of the baseline log step count, the log of the year 1 step counts, and the log of the year 2 step counts from the other days of the week. The year 2 daily log step count is then updated. This is then repeated ten times. We note that, in the imputation of year 1 and year 2 log step counts, instead of including daily logged step counts at baseline, we include their average $\overline {\log ({y}_{i,0,.}^{l}) }$ to reduce noise that may be introduced in the model.

For arm *l*, *l*∈{1,2,3} to impute the baseline logged step count for patient *i* on day *q*, we assume the following model:

6 $$ {\displaystyle \begin{array}{cc}\log \left(\underset{i,0,q}{\overset{l}{y}}\right)=& \alpha +\sum \limits_{k,k\ne q}{\beta}_k\log \left(\underset{i,0,k}{\overset{l}{y}}\right)+\sum \limits_k{\gamma}_k\log \left(\underset{i,1,k}{\overset{l}{y}}\right)\\ {}+\sum \limits_k{\delta}_k\log \left(\underset{i,2,k}{\overset{l}{y}}\right)\\ {}+{\kappa}_1{\mathrm{female}}_i+{\kappa}_2{\mathrm{age}}_i+{\kappa}_3b{1}_i+{\kappa}_4b{2}_i+\dots \\ {}+{\kappa}_{14}b1{1}_i\\ {}+{\kappa}_{15}{\mathrm{BMI}}_i+{\kappa}_{16}{\mathrm{temp}}_{i0q}+{\kappa}_{17}{\mathrm{precip}}_{i0q}\\ {}+{\kappa}_{18}{\mathrm{sun}}_{i0q}\\ {}+{\kappa}_{19}{\mathrm{daylength}}_{i0q}+{e}_{i,0}.\end{array}} $$

To impute the logged step count in year 1 for patient *i* on day *q*, we assume the following model:

7 $$ {\displaystyle \begin{array}{cc}\log \left(\underset{i,1,q}{\overset{l}{y}}\right)=& \alpha +\beta \overline{\log \left(\underset{i,0,.}{\overset{l}{y}}\right)}+\sum \limits_{k,k\ne q}{\gamma}_k\log \left(\underset{i,1,k}{\overset{l}{y}}\right)\\ {}+\sum \limits_k{\delta}_k\log \left(\underset{i,2,k}{\overset{l}{y}}\right)\\ {}+{\kappa}_1{\mathrm{female}}_i+{\kappa}_2{\mathrm{age}}_i+{\kappa}_3b{1}_i+{\kappa}_4b{2}_i\\ {}+\dots +{\kappa}_{14}b1{1}_i\\ {}+{\kappa}_{15}{\mathrm{BMI}}_i+{\kappa}_{16}{\mathrm{temp}}_{i1q}+{\kappa}_{17}{\mathrm{precip}}_{i1q}\\ {}+{\kappa}_{18}{\mathrm{sun}}_{i1q}\\ {}+{\kappa}_{19}{\mathrm{daylength}}_{i1q}+{e}_{i,1}.\end{array}} $$

To impute the logged step count in year 2 for patient *i* on day *q*, we assume the following model:

8 $$ {\displaystyle \begin{array}{cc}\log \left(\underset{i,2,q}{\overset{l}{y}}\right)=& \alpha +\beta \overline{\log \left(\underset{i,0,.}{\overset{l}{y}}\right)}+\sum \limits_k{\gamma}_k\log \left(\underset{i,1,k}{\overset{l}{y}}\right)\\ {}+\sum \limits_{k,k\ne q}{\delta}_k\log \left(\underset{i,2,k}{\overset{l}{y}}\right)\\ {}+{\kappa}_1{\mathrm{female}}_i+{\kappa}_2{\mathrm{age}}_i+{\kappa}_3b{1}_i+{\kappa}_4b{2}_i\\ {}+\dots +{\kappa}_{14}b1{1}_i\\ {}+{\kappa}_{15}{\mathrm{BMI}}_i+{\kappa}_{16}{\mathrm{temp}}_{i2q}+{\kappa}_{17}{\mathrm{precip}}_{i2q}\\ {}+{\kappa}_{18}{\mathrm{sun}}_{i2q}\\ {}+{\kappa}_{19}{\mathrm{daylength}}_{i2q}+{e}_{i,2},\end{array}} $$

and we assume that the residuals are normally distributed: $e_{i, j} \mathbb {I}(treat_{i}=l) \sim N \left (0, \sigma _{j,l} \right)$ for *l*∈{1,2,3} and *j*∈{0,1,2}.

We set the number of imputations *M* to 20 and the number of cycles (burn-in) to 10. After the daily logged step counts are imputed, they are exponentiated and averaged across each week, so that we have 20 complete data sets. The analysis model from Eq. 2 is fitted to each of the 20 complete data sets and then combined using Rubin’s rules.

### Results for the MOVE-IT trial

**Fig. 4:**
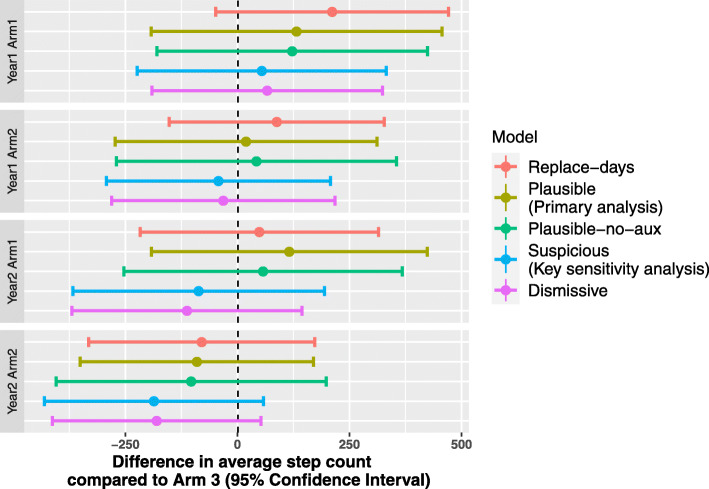
Forest plot showing estimates for the difference in average step count per week for arms 1 and 2 compared to arm 3 for year 1 and 2 for several different choices of models

We considered five possible sets of assumptions for the missing data: the *Plausible* approach which is our preferred model for the primary analysis, the *Suspicious* model which is the key sensitivity analysis, and three further sensitivity analyses: the *Plausible* model without auxiliary variables; the *Replace-days* approach which retains the most amount of data, but involves the use of day-substitution which may not be appropriate in this setting; and finally, the *Dismissive* model which does not impute partially observed data as censored data and assumes that the step counts are MNAR. In Fig. 4, we display the 95% confidence intervals for the difference in average step count for each intervention compared to usual care (arm 3) for year 1 and year 2. Overall, neither the motivational interviewing or group therapy are shown to be effective under the five possible missing data assumptions. However, we note that the there are noticeable differences in the point estimates and variances of the contrasts which reveal the potentially large impact that missing data assumptions can have on the results of the primary analysis.

**Fig. 5:**
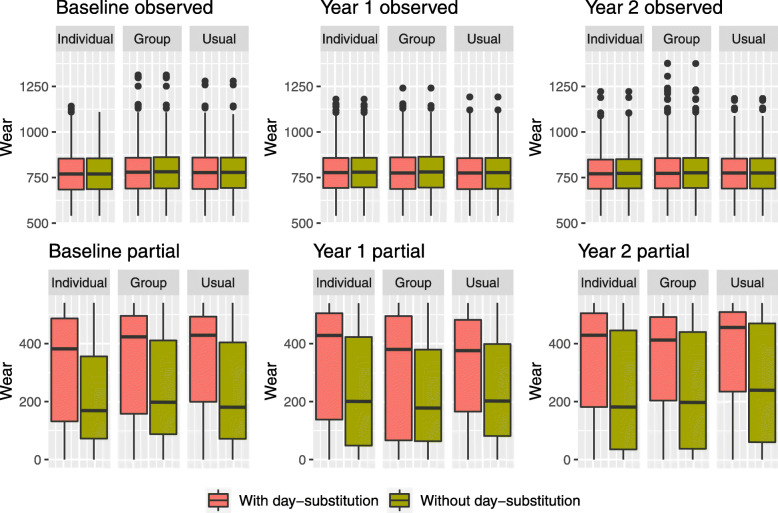
Boxplots showing the distribution of wear time for observed step counts (top) and wear time for partially observed step counts (bottom) at baseline, year 1 and year 2 when day-substitution is carried out across weeks (red) and when it is not carried out (green)

We observe that the point estimate of the contrast is higher for year 1 than year 2 within each model, and the point estimate is higher for arm 1 than arm 2 within each model. We first examine how the missing data approaches impact the point estimates of the contrasts. The *Replace-days* assumption leads to estimates of the contrast that are higher than the other assumptions for year 1. However, this increase may be because the assumption of exchangeability of step counts across weeks, given the day of the week, may not be appropriate as it appears that participants’ wear times are higher outside of the measurement period for partially observed days, as shown in Fig. 5. The point estimates using the *Plausible* assumption appear to change only slightly when auxiliary variables are omitted in the imputation model. We observe that, as expected, the models that assume MAR (*Replace-days*, *Plausible*, *Plausible-no-aux*) lead to higher estimates of the contrasts compared to the models that assume MNAR (*Suspicious* and *Dismissive*).

Next, we examine how the missing data approaches impact the variances of the estimates of the contrasts. *Replace-days* leads to the most precise estimates of the contrast; this is due to the fact that day-substitution has been carried out which leads to a smaller proportion of missing and partially missing observations, as shown in Fig. 2. Comparing the *Plausible* model with the *Plausible-no-aux* model, we observe that the addition of auxiliary variables generally leads to wider confidence intervals. Omitting these auxiliary variables may lead to an underestimate of the variation due to, for example, variation related to weather. In general, we recommend the addition of auxiliary variables to the imputation model as they lead to increased plausibility of the MAR assumption. The models that assume MAR lead to higher variances in the estimates of the contrasts compared to the models that assume MNAR.

In Table 2, we display the estimates of the fixed effects for the primary analysis under the five different models for the missing data. Across the five models, the following variables are significant predictors of step count in year 1 and year 2: sex; age; average step count at baseline and the interaction between average step count at baseline and year. Taking the *Plausible* model as an example, the expected step count is 421.4 steps lower for females than males, holding all other variables constant. The expected step count drops by 40.84 steps per year increase in age, holding all other variables constant, for this older group of participants where the mean age of the participants is 69.75 years [3]. A one-unit increase in baseline step count leads to an increase of 0.782 for steps in year 1, and 0.731 steps in year 2, holding all variables other than year and baseline step count constant. For the *Suspicious* and *Dismissive* models which assume MNAR, the effect of arm 1 is lower than for the other three models.

**Table 2:** Fixed effects of the analysis models where the imputation has been conducted under the *Replace-days, Plausible, Suspicious* and *Dismissive* assumptions for the missing data

	*Replace-days*		*Plausible*		*Plausible-no-aux*		*Suspicious*		*Dismissive*	
			(Primary analysis)				(Key sensitivity analysis)			
arm1	211.4	(1.60)	131.9	(0.81)	122.3	(0.80)	54.44	(0.39)	66.44	(0.51)
arm2	87.91	(0.72)	19.26	(0.13)	42.49	(0.27)	− 42.30	(− 0.33)	− 31.46	(− 0.25)
year2	201.3	(1.26)	10.43	(0.05)	27.90	(0.14)	146.0	(0.90)	87.21	(0.61)
arm1 year2	− 162.5	(− 1.11)	− 16.08	(− 0.10)	− 65.06	(− 0.41)	− 141.0	(− 1.00)	− 178.9	(− 1.38)
arm2 year2	− 167.6	(− 1.32)	− 109.6	(− 0.78)	− 145.6	(− 1.01)	− 143.9	(− 1.15)	− 148.5	(− 1.28)
$\bar {y}_{i,0,.}$	0.804***	(40.75)	0.782***	(28.83)	0.781***	(35.09)	0.729***	(35.28)	0.728***	(35.20)
year2 $\bar {y}_{i,0,.}$	− 0.0597***	(− 3.08)	− 0.0509*	(− 1.86)	− 0.0518*	(− 1.97)	− 0.0560**	(− 2.58)	− 0.0449**	(− 2.26)
female	− 424.1***	(− 3.13)	− 421.4***	(− 2.85)	− 395.5***	(− 2.63)	− 432.0***	(− 3.23)	− 403.0***	(− 3.12)
age	− 31.87***	(− 2.70)	− 40.84***	(− 3.23)	− 43.11***	(− 3.45)	− 28.73**	(− 2.46)	− 28.84**	(− 2.47)
b2	104.4	(0.49)	44.31	(0.19)	102.9	(0.44)	336.7*	(1.67)	331.6	(1.63)
b3	− 30.92	(− 0.12)	− 235.1	(− 0.88)	− 241.9	(− 0.92)	29.22	(0.13)	34.00	(0.15)
b4	276.1	(0.92)	176.9	(0.59)	204.2	(0.58)	315.8	(1.02)	288.4	(1.03)
b5	− 72.39	(− 0.33)	− 205.3	(− 0.79)	− 190.0	(− 0.83)	11.70	(0.05)	50.39	(0.23)
b6	179.9	(0.53)	57.69	(0.16)	110.0	(0.31)	215.6	(0.68)	153.5	(0.48)
b7	51.05	(0.22)	3.919	(0.01)	12.77	(0.05)	215.1	(0.98)	212.5	(0.93)
b8	300.2	(0.84)	374.6	(1.06)	387.8	(1.06)	684.1**	(2.24)	686.4**	(2.21)
b9	534.8	(1.60)	430.8	(1.32)	396.5	(1.09)	463.0	(1.55)	489.6	(1.57)
b10	− 55.07	(− 0.20)	− 120.4	(− 0.44)	0.233	(0.00)	267.6	(1.01)	251.4	(0.97)
b11	93.24	(0.34)	− 142.0	(− 0.46)	− 177.4	(− 0.61)	121.0	(0.46)	156.0	(0.60)
intercept	1239.9***	(5.10)	1592.1***	(5.28)	1561.6***	(5.76)	1261.7***	(5.12)	1237.2***	(5.36)
*N*	3462		3462		3484		3462		3462	

All imputations include auxiliary variables, except for the Plausible model with no auxiliary variables. Values of t-statistics are given in parentheses

The estimates of the random effects, and further details of the results of this analysis, are shown in the “MOVE-IT trial analysis: further results” section.

## Discussion

In this paper, we set out to address a need to create guidelines for analysing accelerometer outcome data with missing values from clinical trials. There are several challenges to consider: firstly, the primary analysis typically has as the outcome the average number of steps over a measurement period, and these averaged step counts are likely to be MNAR, given the observed variables in the primary analysis. Secondly, missingness for step counts is typically defined on the day level, which is a different measurement level to what we use in the primary analysis. Thirdly, some step count observations are partially observed, so there is a need to incorporate bounded below observations in the analysis.

**Table 3:** Random effects of the analysis models where the imputation has been conducted under the *Replace-days, Plausible, Suspicious and Dismissive* assumptions for the missing data

	*Replace-days*	*Plausible*	*Plausible-no-aux*	*Suspicious*	*Dismissive*
		(Primary analysis)		(Key sensitivity analysis)	
**Arm 1**	$\left [\begin {array}{ll}2052.8 & 0.467 \\ \\ 0.467 & 1877.7\\ \end {array}\right ]$	$\left [\begin {array}{ll}2144.2 & 0.443 \\ \\ 0.443 & 2096.1 \\ \end {array}\right ]$	$\left [\begin {array}{ll}2130.9 & 0.461 \\ \\ 0.461 & 2052.7 \\ \end {array}\right ]$	$\left [\begin {array}{ll}2086.3 & 0.466 \\ \\ 0.466 & 2016.2 \\ \end {array}\right ]$	$\left [\begin {array}{ll}2092.6 & 0.448 \\ \\ 0.448 & 2000.9\\ \end {array}\right ]$
**Arm 2**	$\left [\begin {array}{ll} 2134.6 & 0.521 \\ \\ 0.521 & 2096.7\\ \end {array}\right ]$	$\left [\begin {array}{ll}2247.7& 0.496\\ \\ 0.496 & 2128.0\\ \end {array}\right ]$	$\left [\begin {array}{ll}2253.4 & 0.508\\ \\0.508 & 2095.5 \\ \end {array}\right ]$	$\left [\begin {array}{ll}2155.0 & 0.564 \\ \\ 0.564 & 2000.8\\ \end {array}\right ]$	$\left [\begin {array}{ll}2154.2 & 0.559 \\ \\ 0.559 & 1988.5\\ \end {array}\right ]$
**Arm 3**	$\left [\begin {array}{ll}1802.2 & 0.543 \\ \\0.543 & 1900.1\\ \end {array}\right ]$	$\left [\begin {array}{ll}1841.3 &0.550 \\ \\0.550 & 1980.8 \\ \end {array}\right ]$	$\left [\begin {array}{ll}1861.3 & 0.539 \\ \\ 0.539 & 2001.5 \\ \end {array}\right ]$	$\left [\begin {array}{ll}1850.0 & 0.550 \\ \\ 0.550 & 1918.7 \\ \end {array}\right ]$	$\left [\begin {array}{ll}1812.9 & 0.555 \\ \\ 0.555 & 1924.0 \\ \end {array}\right ]$

All imputations include auxiliary variables, except for the *Plausible* model with no auxiliary variables

We propose a framework for identifying the key decisions and assumptions needed for handling missing daily step counts. Multiple Imputation can then be used to impute day-level step counts. MI allows for the separation of the imputation and analysis models, so we can have an imputation model for daily step counts and a primary analysis model for averaged step counts over the measurement period. The imputation model is flexible and can allow for Tobit regression, so that censored (bounded below) observations can be incorporated, and further, auxiliary variables on the day level, such as day of the week and weather, can be included to strengthen the assumption that day-level step counts are MAR given the variables in the imputation model. MI also allows for the relatively simple use of sensitivity analysis to explore a range of missingness assumptions.

Our framework elucidates a number of key questions that need to be answered in order to obtain an appropriate imputation model. Such questions include: should missing step counts be replaced with step counts from outside of the measurement period? Should partially observed step counts be treated as censored data, or treated as completely missing data? Are missing and partially missing data MAR, given the variables in the imputation model? We illustrate sets of possible answers to these questions which constitute an approach for the primary analysis, as well as possible ways to conduct sensitivity analyses. While we focused on primary analysis models where the average step count over the measurement period is the response, our framework is also applicable to analysis models for baseline adjusted daily step counts.

There are a number ways in which this work could be extended. Firstly, we note that step count is only one of several accelerometer outcomes. Modern accelerometers also typically measure time spent in the three types of activity levels: moderate-to-vigorous physical activity (MVPA), light physical activity (LPA) and sedentary behaviour (SB). Time spent in MPVA is a common accelerometer outcome in exercise studies. Many aspects of our framework, such as defining observed, partially observed and missing days using wear time, and the potential use of day-substitution and auxiliary variables, would readily apply to the time spent in MVPA outcome. The imputation approach for right-censored observations would need to be adapted so that the upper bound is defined appropriately for time spent in MVPA. Secondly, the extraction of epoch-level data could lead to a more detailed exploration of the missing data mechanism. Epoch-level data can be used to investigate the times of day when participants are active, which could provide further insight into whether the MAR assumption is plausible for partially observed days. Taking this approach, the cut-off to distinguish between observed and partially observed days could be whether wear time between, say, 9AM and 6PM, is at least 540 min, rather than whether wear time over the entire day is at least 540 min. This would remove a slight ambiguity in the current approach. Thirdly, our exploration is specific to step count data. Rapid developments in technology have allowed wearable devices to measure a plethora of health-related quantities such as gait, body temperature, and blood oxygen saturation [36]. Mobile phones are increasingly being used for behaviour change interventions; see, for example, a review by Oikonomidi et al. (2019) [37]. The problem of missing data will pertain to this vast range of outcomes in wearables. There is thus a need to expand our framework to allow for a range of data types in the outcome, as well as a range of analysis approaches.

We have developed a framework for analysing data from trials with accelerometer outcome measures, and illustrated this with the MOVE-IT trial. Our results show that the point estimates and variances for the contrasts are considerably affected by the missing data assumptions and the presence of auxiliary variables in the imputation model. While, in this particular trial, the overall conclusion of the effectiveness of the intervention remains unchanged, more generally a careful elucidation of the missing data through our framework may lead to different conclusions. We therefore advocate the use of our framework in designing and analysing future studies.

**Fig. 6:**
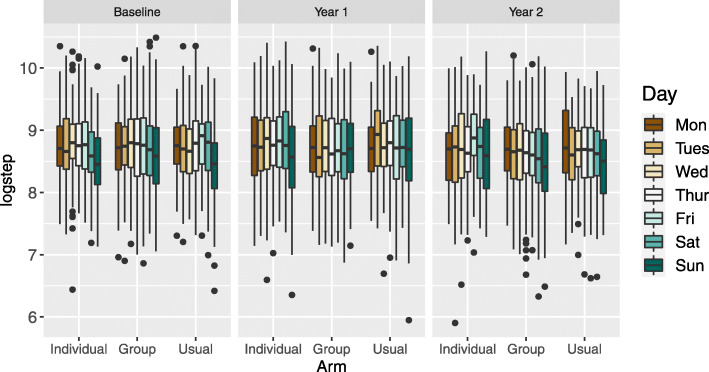
For the *Plausible* model: distribution of the imputed logged step counts for each combination of day of the week, year and arm. Imputed logged step counts for *M*=1 are shown

**Fig. 7:**
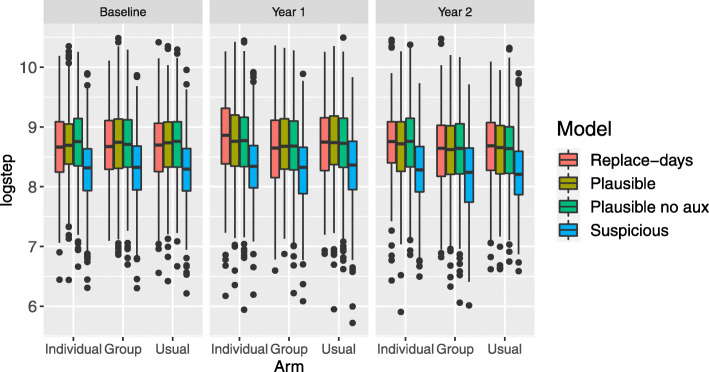
Distribution of the imputed partially observed logged step counts for each combination of year and arm. Imputed logged step counts for *M*=1 are shown for the four models considered

## Appendix

### Wear time

To investigate whether the distribution of wear time is affected by day-substitution, we display boxplots showing the distribution of wear time for complete observations in the top row and wear time for partial observations (bottom row) for each year when the day-substitution is carried out (in red) and when it is not carried out (in green) in Fig. 5. For complete observations, the distribution of wear time appears unaffected by day-substitution; the median wear time is approximately 750 min or 12.5 h. For partially observed step counts, however, it appears that the average wear time is longer when day-substitution is carried out, suggesting that the exchangeability assumption across weeks given the day of the week may not be plausible in this setting.

**Fig. 8:**
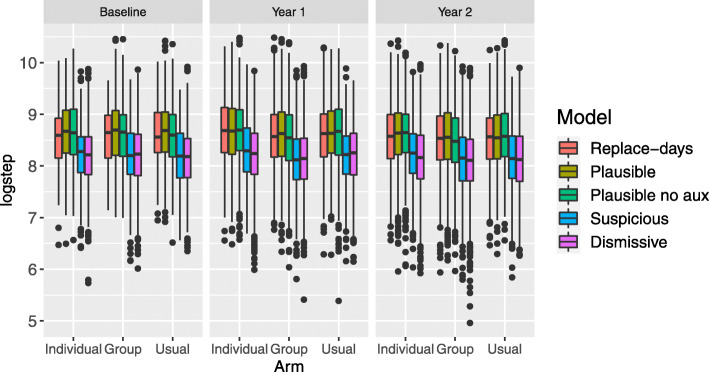
Distribution of the imputed missing logged step counts for each combination of year and arm. Logged step counts for *M*=1 are shown for the five models considered

### Obtaining weather data

#### Temperature

We used daily temperature data from the Met Office [38]. We used data from the Heathrow weather station and obtained the maximum and minimum air temperature during the day (9:00–21:00).

#### Rainfall

We used rainfall data from the Met Office [39]. We used data from the Heathrow weather station (station ID: 708) for 2016 and 2017. For the years 2013–2015, data from Heathrow station is incomplete, so we used CHEAM P STA NO 2 (station ID: 6586), which is located near Sutton. We obtained the total rainfall per day (for the 24-h period) in mm.

Since rainfall in mm was seen to be highly right-skewed, we took the square root transformation.

#### Sunshine

We obtained data on daily duration of sunshine from the Met Office [40]. We used data from Heathrow station (station ID: 708). Sunshine duration is measured in hours. There were 4 days in 2015 with missing observations, and 3 days in 2017 with missing observations. Observations from Wattisham weather station in Suffolk (Station ID: 440) were used for these days.

#### Length of day

Length of day can be calculated by solar orbital geometry. The daylength function in the R package geomsphere [41] is used to compute daylength (photoperiod) for a given latitude and date. We used the latitute for Bromley (51.4).

There were other weather variables available, such as snowfall, hail, gale day, but these variables had a high proportion of zeros. We did not use them as auxiliary variables in our analysis.

### MOVE-IT trial analysis: further results

We show further results from the analysis of the MOVE-IT trial with the MI framework in this section.

#### Random effects

We display the random effects for the primary analysis model in Table 3. The variances for the residuals are highest for individuals taking group therapy (arm 2), both for year 1 and year 2, followed by the variances for the residuals for those in usual care (arm 3). However, the variance for the residuals in group therapy may be inflated because there is a clustering of individuals who were in the same therapy group which is not reflected in the model, as it led to the model not converging. The covariances between the observations in year 1 and year 2 is lowest for arm 1.

#### Imputed step counts

We look at the distributions of the imputed logged step counts. In Fig. 6, we display boxplots of the imputed logged step counts for each combination of day of the week, year and arm for the *Plausible* approach. We observe that there is a weekend effect; the step counts for Sunday, in particular, are generally lower than those of weekdays. This effect is present across arms and also across years.

We display the distribution of the imputed partially observed logged step counts for each combination of year and arm in Fig. 7 for *M*=1. The boxplots are shown for the four approaches which incorporate partially observed data (the *Dismissive* approach does not). In Fig. 8, we plot the boxplots for missing data for all five models. Corresponding to the Forest plot in Fig. 4, we observe that the imputed logged step counts are lower for the *Suspicious* and *Dismissive* models.

## Supplementary Information

**Additional file 1** The STATA DO-File (.do) provides example code for using multiple imputation with interval regression.

## Data Availability

Data are available on reasonable request. Declarations

## Abbreviations

EM: Expectation-maximization
MAR: Missing at random
MCAR: Missing completely at random
MI: Multiple imputation
MNAR: Missing not at random
